# High yield expression of leptospirosis vaccine candidates LigA and LipL32 in the methylotrophic yeast *Pichia pastoris*

**DOI:** 10.1186/1475-2859-9-98

**Published:** 2010-12-06

**Authors:** Daiane D Hartwig, Thaís L Oliveira, Fabiana K Seixas, Karine M Forster, Caroline Rizzi, Cláudia P Hartleben, Alan JA McBride, Odir A Dellagostin

**Affiliations:** 1 Núcleo de Biotecnologia, Centro de Desenvolvimento Tecnológico, Universidade Federal de Pelotas, Pelotas, RS, Brazil; 2 Laboratório de Patologia e Biologia Molecular, Instituto Gonçalo Moniz, Fiocruz-BA, Salvador, BA, Brazil

## Abstract

**Background:**

Leptospirosis, a zoonosis caused by *Leptospira *spp., is recognized as an emergent infectious disease. Due to the lack of adequate diagnostic tools, vaccines are an attractive intervention strategy. Recombinant proteins produced in *Escherichia coli *have demonstrated promising results, albeit with variable efficacy. *Pichia pastoris *is an alternative host with several advantages for the production of recombinant proteins.

**Results:**

The vaccine candidates LigANI and LipL32 were cloned and expressed in *P. pastoris *as secreted proteins. Large-scale expression resulted in a yield of 276 mg/L for LigANI and 285 mg/L for LipL32. The recombinant proteins were glycosylated and were recognized by antibodies present in the sera of patients with severe leptospirosis.

**Conclusions:**

The expression of LigANI and LipL32 in *P. pastoris *resulted in a significant increase in yield compared to expression in *E. coli*. In addition, the proteins were secreted, allowing for easy purification, and retained the antigenic characteristics of the native proteins, demonstrating their potential application as subunit vaccine candidates.

## Background

*Leptospira interrogans sensu lato *is the causative agent of Leptospirosis, one of the most widespread zoonotic diseases in the world [1-3]. In Brazil alone there are over 10,000 cases of leptospirosis reported annually during the epidemics that affect the poor communities in the major urban centres of Brazil [4]. Mortality ranges from 10-15% in cases of the traditional Weil's disease and can be over 70% in cases of severe pulmonary haemorrhage syndrome (SPHS) and, even with aggressive intervention strategies, mortality remains high [5-7]. Due to the lack of adequate tools leptospirosis is under-diagnosed, therefore vaccination remains a viable alternative for the management of this disease. Several groups, including our own, have demonstrated the use of subunit vaccines against leptospirosis, albeit with varying degrees of efficacy [8-10], in particular the use of the Leptospiral immunoglobulin-like (Lig) proteins, LigA and LigB [11-14], and the immunodominant lipoprotein, LipL32 [15-18].

*Escherichia coli *has been used extensively as a host for heterologous protein expression, but potential limitations include the yield, folding and post-translational modifications of the recombinant protein. An alternative host to *E. coli *is the methylotrophic yeast, *Pichia pastoris*. This yeast strain has emerged as a powerful and inexpensive expression system for the heterologous production of recombinant proteins with the following characteristics: (i) techniques for genetic modifications are available; (ii) proteins may be secreted; (iii) post-translational modification and (iv) high yield, reviewed in [19-21].

We previously expressed the Lig polypeptides, LigANI, LigBNI and LigBrep, in several *E. coli*-based expression systems. To date the recombinant proteins were insoluble, required extensive dialysis during purification and the yield was poor [13]. In this work we describe the use of the methylotrophic yeast *P. pastoris *for the cloning, expression, purification and antigenic characterization of the leptospiral vaccine candidates LigANI and LipL32.

## Results

### Plasmid construction and sequence analysis

**Figure 1:**
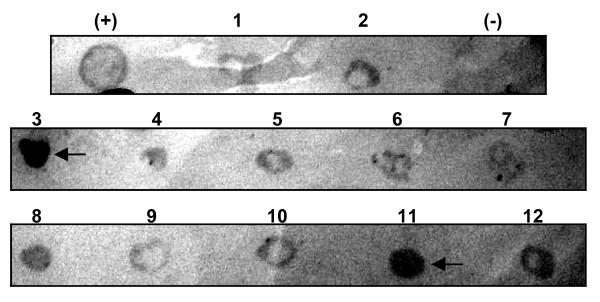
**Screening for *P. pastoris *recombinant clones expressing rLigANI and rLipL32**. Colony blot analysis of transformed *P. pastoris *strain KM71 H with anti-6×His Mab. The *t*gD recombinant protein expressed in *P. pastoris *KM71 H was the positive control (+) and untransformed *P. pastoris *KM71 H was the negative control (-). Spots 1-7 are representative rLigANI colonies and 8-12 are representative rLipL32 colonies. Arrows indicate the colonies that were selected for large-scale expression studies.

The DNA sequences that encode for the LigA polypeptide, LigANI, (1800 bp) and LipL32 (766 bp) were amplified by PCR and cloned into the *P. pastoris *expression vector pPICZαB. Of the 150 *P. pastoris *colonies screened for expression of each recombinant protein, 30 colonies were strongly recognised by a monoclonal antibody (Mab) specific to the 6×His tag at the C-terminus of the recombinant proteins. Colony PCR was used to confirm the presence of the insert in the expression vector and clones exhibiting the highest expression levels were selected for further expression studies, Figure 1.

### Expression of LigANI and LipL32 in *P. pastoris*

**Figure 2:**
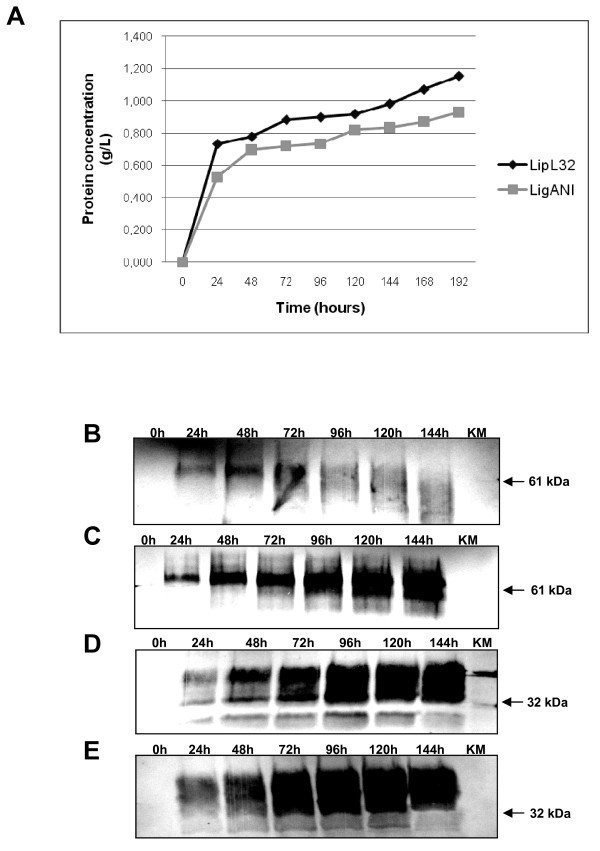
**Expression of rLigANI and rLipL32 proteins in *P. pastoris***. Time courses for the expression of secreted rLigANI and rLipL32 by *P. pastoris *induced for up to 192 hours (8 days), (A) as determined by protein concentration (mg/mL). Western blot analysis of the intracellular (pellet) and secreted (supernatant) expression of rLigANI (B and C, respectively) and rLipL32 (D and E, respectively), using polyclonal anti-LigANI sera or anti-LipL32 Mab. Samples (cells and supernatant) were collected at the various hourly time points indicated. KM - negative control: untransformed *P. pastoris *KM71 H culture.

The coding sequences for the recombinant proteins LigANI (rLigANI) and LipL32 (rLipL32) cloned in pPICZαB were under the control of the *AOX1 *promoter. In addition, pPICZαB contains the α-factor signal sequence from *S. cerevisiae*, allowing secretion of the recombinant protein. The concentration of rLigANI and rLipL32 in the culture supernatant was found to increase with time, Figure 2A, and is related with a decrease in the intracellular concentration of rLigANI, Figure 2B and 2C. In contrast, while the secretion of rLipL32 increased, so did the intracellular concentration, Figure 2D and 2E. Recombinant proteins of the expected size were observed, rLigANI (61 kDa) and rLipL32 (32 kDa), yet there was evidence of larger proteins, suggesting that the recombinant proteins had been glycosylated by *P. pastoris*. Following 196 h induction at 28°C, the concentration of secreted protein reached 0.93 g/L and 1.2 g/L for rLigANI and rLipL32, respectively. Large-scale (2 L cultures) expression of rLigANI and rLipL32 resulted in yields of 276 mg/L and 285 mg/L, respectively.

### Recombinant protein purification and concentration

**Figure 3:**
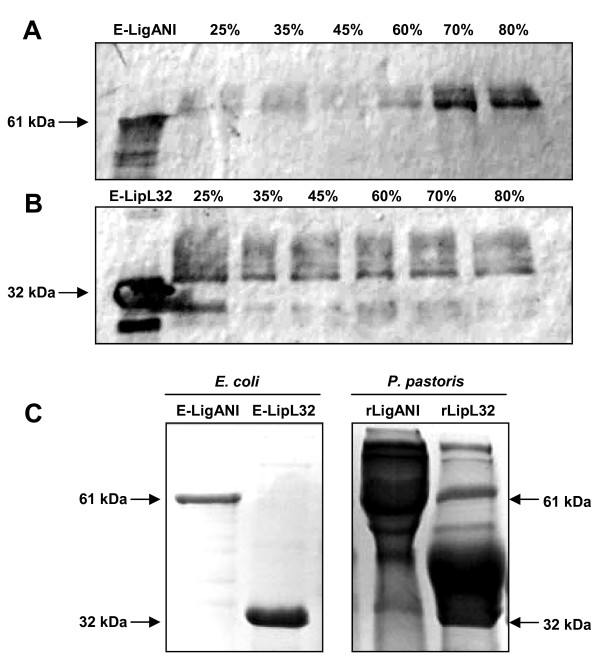
**Purification of rLigANI and rLipL32 expressed in *P. pastoris***. Recombinant proteins purified by precipitation with ammonium sulphate or by ultrafiltration. Ammonium sulphate precipitated proteins were detected by Western blotting with (A) polyclonal anti-LigANI sera or (B) an anti-LipL32 Mab. The effect of the various concentrations of ammonium sulphate (expressed as percentage values) on the precipitation of the recombinant proteins is displayed. (C) Affinity chromatography purified recombinant LigANI (61 kDa) and LipL32 (32 kDa) produced in *E. coli *compared to purification by ultrafiltration of rLigANI and rLipL32 secreted by *P. pastoris*. An equal volume (10 μL) of both proteins was loaded on the gel.

The supernatant containing the secreted rLigANI and rLipL32 was collected and purified/concentrated using three alternative methods. In the first method, the proteins were purified by ammonium sulphate precipitation. The optimal salt concentration for rLigANI was 70-80%, while the precipitation of rLipL32 was similar under all concentrations tested. The recombinant proteins were dialyzed to remove the ammonium sulphate and then analysed by Western blotting, Figure 3A, B. Once again, there was evidence of post-translation modification of the recombinant proteins. The yield for both rLigANI and rLipL32 was similar, approximately 70 mg/L, corresponding to 24.5 and 27.6% of total protein, respectively. In the second method, the supernatant was concentrated by ultrafiltration which reduced the starting volume by 97%. The yield for rLigANI was 183 mg/L (66.3% total protein) compared to 106 mg/L (37.3% total protein) for rLipL32. The samples were observed by 12% SDS-PAGE and compared to recombinant proteins expressed and purified from *E. coli *(Figure 3C). In the third method, the secreted proteins were concentrated by lyophilisation. There was a 10-fold reduction in the initial sample volume and the yield was 239 mg/L rLigANI and 224 mg/L rLipL32, equivalent to 86.7 and 70.7% total protein, respectively.

### Deglycosylation of LigANI and LipL32

**Figure 4:**
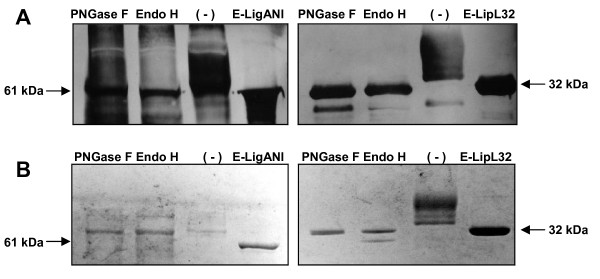
**Deglycosylation of rLigANI and rLipL32 produced by *P pastoris***. To evaluate the post-translational modification of the rLigANI and rLipL32 proteins produced and secreted by *P. pastoris*, the proteins were deglycosylated with PNGase F and Endo H. The resultant proteins were visualized by (A) Western blotting with polyclonal anti-LigANI sera and an anti-LipL32 Mab or by (B) SDS-PAGE stained with Coomassie blue. The proteins were digested with PNGase F, Endo H or without enzyme (-). E-LigANI (61 kDa) and E-LipL32 (32 kDa) recombinant proteins were expressed and purified from *E. coli*.

In an analysis, using Vector NTI Advance 10.0 (Invitrogen) software, of the recombinant protein amino acid sequences, LigANI was found to have seven potential N-glycosylation sites, compared to one for LipL32. N-Glycosidase F (PNGase F) removes oligomannose, hybrid, and complex N-glycans attached to asparagine, **while **Endoglycosidase H (Endo H) releases oligomannose and hybrid N-glycans, but not complex N-glycans, and **were used to deglycosylate **the recombinant proteins. Following deglycosylation, the larger molecular weight species were no longer evident and the size of the rLigANI and rLipL32 corresponded to the equivalent protein produced in *E. coli*, Figure 4. There did not appear to be any difference in action between the two enzymes used.

### Antigenicity of the recombinant LigANI and LipL32 proteins

**Figure 5:**
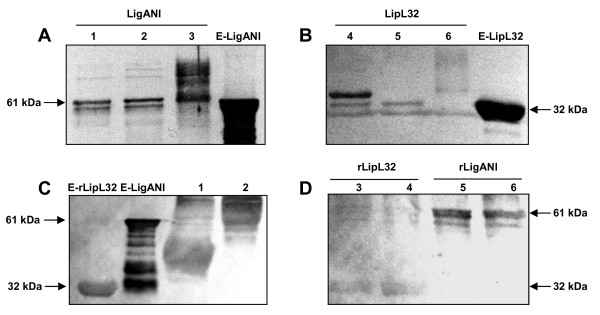
**Antigenicity of the various forms of rLigANI and rLipL32**. Antigenicity was evaluated using rabbit anti-*Leptospira *sera (A) Lanes: 1 - rLigANI + PNGase F; 2 - rLigANI + Endo H; 3 - glycosylated rLigANI and (B) Lanes: 4 - rLipL32 + PNGase F; 5 - rLipL32 + Endo H; 6 - glycosylated rLipL32 or (C) convalescent sera from leptospirosis patients, Lanes: 1 - glycosylated rLipL32; 2 - glycosylated rLigANI and (D) 3 - rLipL32 + PNGAse F; 4 - LipL32 + Endo H; 5 - rLigANI + PNGAse F; 6 - rLigANI + Endo H. E-LigANI (61 kDa) and E-LipL32 (32 kDa) recombinant proteins were expressed and purified from *E. coli*.

The antigenicity of the purified proteins was evaluated by Western blotting with sera from leptospirosis patients and with rabbit anti-*Leptospira *hyperimmune sera. The recombinant proteins LigANI and LipL32 produced in *E. coli *were included as positive controls. Both glycosylated and deglycosylated (Endo H and PNGase F treated) rLigANI were recognised by the human and rabbit immune sera, Figure 5A, C and 5D, as were the glycosylated and deglycosylated forms of rLipL32, Figure 5B, C and 5D.

## Discussion

Previous studies have demonstrated the use of the Lig proteins and LipL32 in a range of formats, including recombinant proteins [11-14], DNA vaccines [17,22], microspheres and liposomes [23,24], fused to a cholera toxin subunit [25] or expressed in *M. bovis *bacille Calmette-Guérin [16]. However, vaccine efficacy in the animal models has been highly variable for these and other *Leptospira *proteins and they do not induce sterilizing immunity, reviewed in [26]. Several reports suggest that the most likely explanation for the lack of a consistent protective effect with recombinant proteins produced in *E. coli *is the failure of the proteins to fold correctly [13,22]. Structural modelling of Lig molecules predicted that the bacterial immunoglobulin-like (Big) repeat domains have a highly folded β-immunoglobulin sandwich structure [27]. *E. coli *expressed the full-length LigA at very low levels because of its high toxicity, which resulted in a 50-fold decrease in viability of cells [28]. Furthermore, expression of recombinant LigA in the *E. coli *pET expression system failed [14].

*P. pastoris *is an important host organism for the production of recombinant proteins [19]. The large-scale production of recombinant proteins is necessary for pharmaceutical, biomedical and biotechnological applications, therefore it is important to develop and to optimize techniques for increased yield of the proteins of interest. In this work we cloned and expressed a C-terminal fragment of LigA, LigANI, which includes six Big repeat domains of the LigA protein, in the methylotrophic yeast *P. pastoris*. In addition, the full-length LipL32 protein was also expressed as a secreted protein. Previously we reported the expression of recombinant LigANI in *E. coli *with a yield of 6-10 mg/L [13], while recombinant LipL32 was expressed at 40 mg/L [29]. In this study we report that large-scale expression in *P. pastoris *resulted in yields of over 250 mg/L for both rLigANI and rLipL32, without the need for subsequent solubilisation and/or re-folding steps. The strain used in this study, KM71 H, has a deletion in the *AOX1 *gene, which is partly replaced by *ARG4 *from *S. cerevisiae *and the phenotype of these strains is Mut^S ^(Methanol utilization slow). The use of such strains is advantageous as they do not require large amounts of methanol in large-scale cultures [19-21].

Three low-cost purification strategies were evaluated, namely: i) ammonium sulphate precipitation and desalting by dialysis, ii) ultrafiltration and iii) lyophilisation. The most significant results in terms of yield were obtained using lyophilisation and ultrafiltration to purify and/or concentrate the proteins. This is an important observation as these techniques are applicable to large-scale cultures grown in bioreactors on an industrial scale. During ultrafiltration the columns used had a cut-off of 30 kDa and our results demonstrated a decreased yield of the rLipL32 protein, possibly due to the fact that the cut-off is very close to the molecular weight of the recombinant protein. There was a significantly lower yield of both rLigANI and rLipL32 when purified by ammonium sulphate precipitation.

LigANI and LipL32 were predicted to contain potential N-glycosylation sites and treatment of the recombinant proteins with the enzymes Endo H and PNGase F confirmed that post-translational modification had occurred during production and secretion in *P. pastoris*, Figure 4. Deglycosylation removed the N-glycans attached to asparagine and when analysed by SDS-PAGE and Western blotting, rLigANI and rLipL32 had similar molecular weights as the corresponding proteins expressed in *E. coli*. N-glycosylation in yeast has a composition of Man_n_GlcNAc_2 _(Man: Mannose; GlcNAc: N-acetylglucosamine), where *n *is the number of mannose oligosaccharides attached to the structure. This number has been found to vary in *P. pastoris *from 3 to 17, depending on the expressed protein [30,31]. The attachment of a large number of mannose residues, known as hyperglycosylation, is rarely observed in *P. pastoris*, compared to *S. cerevisiae *which hyperglycosylates the majority of expressed proteins. Glycosylation can be influenced by some of the bioprocess parameters used during growth and purification steps [32,33]. Therefore, secreted proteins that are easily recovered from the growth medium are likely to maintain the structure of the recombinant protein. This may improve the protective immune response against leptospirosis when rLigANI and rLipL32 are used as subunit vaccine candidates.

## Conclusions

We believe that this is the first report of the use of *P. pastoris *to express pathogenic *Leptospira *antigens. The aim of the study was to evaluate the large-scale expression of the vaccine candidates LigA and LipL32 proteins in *P. pastoris*. The rLigANI and rLipL32 proteins described in this study were soluble and the purification step used simple and inexpensive methods. Indeed, not only were the proteins expressed at a high level, but they retained the antigenic characteristics of native the proteins. Furthermore, glycosylated rLigANI and rLpiL32 were recognised by the antibodies presents in the sera of leptospirosis patients and with antibodies raised against a heterologous *Leptospira *serovar.

## Methods

### Bacterial strains and growth conditions

*L. interrogans *serovar Copenhageni strain Fiocruz L1-130, originally isolated from a patient with severe leptospirosis [34], was cultivated in Ellinghausen-McCullough-Johnson-Harris (EMJH) medium supplemented with *Leptospira *Enrichment EMJH (Difco, USA) at 30°C. *E. coli *strain TOP10 (Invitrogen) was grown in Luria-Bertani (LB) medium (1% tryptone, 0.5% yeast extract, 0.5% NaCl and 2% agar) at 37°C with the addition of zeocin to 25 μg/mL. *P. pastoris *strain KM71 H (Mut^S^, Invitrogen) was grown in Yeast extract peptone dextrose (YPD) medium (1% yeast extract, 2% peptone and 2% D-glucose) supplemented with 100 μg/mL of zeocin at 28°C.

### Cloning *ligA *and *lipL32*

We previously identified a C-terminal fragment of LigA, LigANI, as a vaccine candidate [13]. Primers to amplify the DNA sequences coding for the LigANI polypeptide and the full-length *lipL32 *gene were designed according the genome sequence of *L. interrogans *serovar Copenhageni strain Fiocruz L1-130 [GenBank: AE016823]. The primer sequences (*Eco*RI and *Kpn*I sites are underlined) used in this study were: *ligANI*_F: 5'-CGGAATTCAATAATGTCTGATATTCTTACCGT, *ligANI*_R: 5'-TAGGTACCATGGCTCCGTTTTAATAGAG and *lipL32*_F: 5'-CGGAATTCTAGGTGGTCTGCCAA, *lipL32*_R: 5'-GGGGTACCACTTAGTCGCGTCA. The PCR products were cloned in-frame into the pPICZαB vector (Invitrogen, Brazil). The identity of the inserts was determined by DNA sequencing using the DYEnamic ET Dye Terminator Cycle Sequencing Kit for MegaBACE DNA Analysis Systems - MegaBACE 500 (GE Healthcare, Brazil). Recombinant plasmids containing the LigANI coding sequence, pPIC-LigANI, and *lipL32*, pPIC-LipL32, were propagated in *E. coli *TOP10, and the plasmids isolated using the Perfectprep Plasmid Maxi kit (Eppendorf, USA). The plasmids were linearized with restriction enzyme *Pme*I (New England BioLabs, USA). The linear plasmid DNA was purified by phenol-chloroform extraction and DNA precipitation. *P. pastoris *competent cells were transformed by electroporation (25 μF, 200 Ω, 2 kV) with 10 μg of linear plasmid DNA.

### Screening for expression of recombinant LigANI and LipL32

Approximately 150 colonies of each plasmid construct were plated onto Buffered methanol-complex medium (BMMY: 1% yeast extract, 2% peptone, 1.34% yeast nitrogen base, 0.00004% biotin, 0.5% methanol, 100 mM potassium phosphate and 2% agar, pH 6.0). Following 24, 48 and 72 h incubation at 28°C, expression of rLigANI and rLipL32 was induced with 1% methanol and evaluated after 96 h. Expression of the recombinant proteins was confirmed by colony immunoblotting [35]. Briefly, a nitrocellulose membrane (Hybond ECL, GE Healthcare) was placed onto the surface of each petri dish and in direct contact with the colonies for 3 h at 28°C. Any adherent matter was removed from the membrane by washing with PBST (PBS, pH 7.4, 0.05% (v/v) Tween 20). After blocking (PBST, 5% non-fat dried milk), the membrane was incubated for 1 h at room temperature with anti-6×His-peroxidase conjugate (Sigma-Aldrich, Brazil) at a dilution of 1:8,000 in PBS. After three washes (5 min each) positive colonies were detected with 4-chloro-1-naphthol (Sigma-Aldrich).

The presence of the PCR products in the recombinant plasmids was also confirmed by colony PCR. Crude genomic DNA extracts were prepared by boiling selected yeast recombinant clones in water. PCR was performed as described above, using the crude genomic DNA extracts as template. PCR products were analysed by horizontal gel electrophoresis and visualized with GelRed (Uniscience, Brazil).

### Expression of LigANI and LipL32 proteins in *P. pastoris *KM71H

A recombinant clone for each construct (rLigANI and rLipL32), positive for expression and colony PCR, was selected and inoculated into a 1 L baffled flask containing 200 mL BMGY broth (differs from BMMY in that the 1% methanol is replaced by 1% glycerol). The cultures were incubated at 28°C, with shaking (250 rpm), for approximately 16-18 h until an OD_600 _of 2 to 6 was reached. The cells were harvested by centrifugation at 3,000 × *g *for 5 min and the cell pellet resuspended in the supernatant equivalent to 1/10 of the original volume (20 mL). The culture was place in a 100 mL baffled flask and return to the incubator. Expression was induced by the addition of methanol to a final concentration of 0.5%. Samples (supernatant and cells) were collected at the following time points: 0, 24, 48, 72, 96, 120, 144, 168 and 196 h and stored at -80°C. The cell pellets were suspended in breaking buffer (50 mM sodium phosphate, 1 mM PMSF, 1 mM EDTA and 5% glycerol) and an equal volume of acid-washed glass beads (0.5 mm Ø). The samples were vortexed for 30 s followed by incubation on ice for 30 s (8 cycles), centrifuged at 16,000 × *g *for 10 min at 4°C and the cleared supernatant stored at -80°C.

The expression of the recombinant proteins were analysed by (12%) sodium dodecyl sulphate-polyacrylamide gel electrophoresis (SDS-PAGE) and visualised by staining with Coomassie Blue or Western blotting (WB). Samples were suspended in loading buffer (2% SDS, 500 mM Tris pH 7.6, 1% bromophenol blue, 50% glycerol and 1% 2-mercaptoethanol) and boiled for 10 min before separation by SDS-PAGE. For the WB assay the proteins were electro transferred to a nitrocellulose membrane (Hybond ECL, GE Healthcare). After blocking, PBS, 5% non-fat dried milk, overnight at 4°C and three washes (5 min per wash) in PBST, the membranes were incubated for 1 h with anti-LipL32 Mab (1:500 in PBS) or mouse anti-LigANI polyclonal (1:500 in PBS), followed by 3 washes (5 min per wash) in PBST. The rabbit anti-mouse IgG peroxidase conjugate (Sigma-Aldrich), diluted 1:6,000 in PBS, was added and incubated for 1 h. The membranes were washed 5× in PBST and the reactions were developed with 4-chloro-1-naphthol (Sigma-Aldrich).

LigANI and LipL32 were produced in large-scale using the *P. pastoris *Mut^S ^secretory phenotype, under the same conditions described above. Briefly, *P. pastoris *was grown in BMGY medium (2 L) to an OD_600 _of 2 to 6, harvested by centrifugation and suspended in 200 mL BMMY expression medium (1/10 of the original culture volume). The expression of the recombinant proteins was induced for 144 h by the addition of methanol to 0.5%. The supernatant containing the secreted recombinant proteins was cleared by centrifugation, and stored at -80°C.

### Purification and concentration of rLigANI and rLipL32

Three different strategies were used to purify and concentrate the secreted recombinant proteins. The first strategy was based on ammonium sulphate precipitation: 85% ammonium sulphate was added to the culture supernatant at 4°C, to final concentrations of: 25, 35, 45, 60, 70 and 80%. The precipitated proteins were collected by centrifugation at 10,000 × *g *for 15 min at 4°C, suspended in PBS and dialyzed in the same buffer for 48 h. Microcon YM-30 Amicon Bioseparation filters (Millipore, USA), 30 kDa cut-off, were used to concentrate the recombinant proteins expressed in the supernatant, following the manufacturer's protocol. Alternatively, proteins were concentrated by lyophilisation (Edwards Micro Modulyo) over 28 h and suspended in PBS, resulting in a 10-fold concentration of the initial sample. The protein concentration in culture supernatants, concentrates and purified protein samples were determined using the BCA Protein Assay Kit (Pierce, USA) with bovine serum albumin (BSA) as a the standard.

### Deglycosylation of rLigANI and rLipL32

Purified rLigANI and rLipL32 (1-20 μg) were incubated with 1× glycoprotein reaction buffer at 100°C for 10 min to completely denature the glycoproteins. Deglycosylation was carried out at 37°C for 1 h with 5× G5 (Endoglycosidase H) or 10× G7 (N-Glycosidase F) reaction buffer and 1-5 μl of the relevant enzyme (Endoglycosidase H or N-Glycosidase F) according to the manufacturer's instructions (New England BioLabs).

### Antigenicity of rLigANI and rLipL32

The ability of the recombinant proteins to interact specifically with products of the immune response was determined by WB using sera collected from leptospirosis patients and hyperimmune sera from infected rabbits. The use of patient sera for these experiments was approved by the Internal Review Board of the Gonçalo Moniz Institute, Fiocruz-BA. A pool of convalescent sera from severe leptospirosis patients was used at a dilution of 1:300 and an anti-human IgG peroxidase conjugate at a 1:2,000 dilution. Rabbit anti-*Leptospira *hyperimmune sera, specific to *L. interrogans *serovar Canicola strain Tande, was used at a dilution of 1:500 and an anti-rabbit IgG peroxidase conjugate at a 1:3,000 dilution.

## Competing interests

AJAM and OAD are inventors on a patent submission entitled: LigA and LigB proteins (Leptospiral Ig-like (Lig) domains) for vaccination and diagnosis (Patent nos. BRPI0505529 and WO 2007070996). The other authors declare no competing interests.

## Authors' contributions

DDH participated in the study design, performed the experiments and in the writing of the manuscript. TLO performed the experiments. FKS participated in the construction of the plasmids. KMF and CPH participated in the experiments on protein antigenicity and CR participated in the protein purification steps. AJAM participated in the data analysis and the writing of the manuscript. OAD coordinated the study and participated in the writing of the manuscript. All authors read and approved the final manuscript.
